# A MIXED-METHODS EVALUATION OF A NURSE-LED INTERPROFESSIONAL INTERVENTION FOR COMMUNITY-DWELLING OLDER ADULTS

**DOI:** 10.1093/geroni/igz038.1084

**Published:** 2019-11-08

**Authors:** Anna-Rae L Montano

**Affiliations:** University of Connecticut, Storrs, Connecticut, United States

## Abstract

This study aims to assess the relationship between an Interprofessional Collaborative Practice (IPCP) intervention for community-dwelling older adults, Geriatric Outreach and Training with Care! (GOT Care!), and the observed 26% reduction in Emergency Department (ED) visits for the 51 older adults who participated. This study utilized a convergent parallel mixed-methods design that included a historical prospective matched cohort study and semi-structured interview surveys. The 51 GOT Care! participants were retrospectively matched to 51 control participants on several variables and ED visits were assessed for each group at 6, 8 and 12 months. Mixed effects generalized linear modeling, with a Poisson response, a log link function, and a random effect for pair, was conducted to analyze the quantitative data. Stakeholders, including hospital administrators and faculty clinicians, of GOT Care! responded to electronic semi-structured interview surveys regarding the relationship between GOT Care! and the observed reduction in ED visits. Content analysis of the responses was completed. The themes from the stakeholder interviews were integrated into the historical prospective matched cohort study to further explore the relationship between GOT Care! and the reduction in ED visits. The results of this study are still pending. This study has implications regarding the utilization of an IPCP model within an academic-practice partnership in the aim of reducing ED visits for vulnerable community-dwelling older adults. Hospital administrators can also utilize the results of this study to gauge the value of IPCP models within their hospital systems.

